# Rabin8 Protein Interacts with GTPase Rheb and Inhibits Phosphorylation of Ser235/Ser236 in Small Ribosomal Subunit Protein S6

**Published:** 2011

**Authors:** А.А. Parkhitko, О.О. Favorova, E.P. Henske

**Affiliations:** Pirogov Russian National Research Medical University; Fox Chase Cancer Center, Philadelphia, USA; Brigham and Women’s Hospital, Harvard Medical School, USA

**Keywords:** complex mTORC1, Rheb, Rabin8, small ribosomal unit protein S6

## Abstract

The mammalian target of rapamycin (mTOR) is a serine/threonine kinase that in association with Raptor, mLST8, PRAS40 and Deptor forms a complex (mTORC1) playing the key role in the regulation of protein biosynthesis, transcription, cellular metabolism, apoptosis and autophagy; mainly via direct phosphorylation of S6 kinases. mTORC1 is activated by growth factors and amino acids via the activation of Rheb GTPase. In the current study, we demonstrate for the first time that the over-expression of Rabin8, which functions as a guanine nucleotide exchange factor for Rab8 GTPase, suppresses phosphorylation of Ser235/Ser236 in ribosomal protein S6. Downregulation of Rabin8 using small interfering RNA (siRNA) increases the phosphorylation of Ser235/Ser236 in ribosomal protein S6. Furthermore, Rabin8 can be immunoprecipitated with Rheb GTPase. These results suggest the existence of a novel mechanism of mTORС1 regulation and its downstream processes. Since Rabin8 is a known regulator of ciliogenesis, a potential link can exist between regulation of Rheb/mTORC1 and ciliogenesis.

## INTRODUCTION

Highly conserved serine/threonine protein kinase mTOR (mammalian target of rapamycin) belongs to the family of phosphatidyl inositol 3’ kinase-related kinases (PIKK) and is the key enzyme of the mTOR-signaling pathway controlling the accumulation of the cell mass in many eukaryotes. mTOR as a catalytic subunit is a component of two hetero-olygomeric complexes, mTORC1 and mTORC2, that have different functions. mTORC1 is a functional dimer containing two subunits of each one of the following proteins: mTOR, Raptor (regulatory associated protein of mTOR), mLST8 (mammalian lethal with sec-13), PRAS40 (proline-rich AKT substrate 40 kDa), and Deptor (DEP-domain-containing mTOR-interacting protein) [1, 2]. mTORC1 phosphorylates a wide range of effectors regulating the processes of protein synthesis, cell proliferation, apoptosis, and autophagy in response to external signals [3, 4]. mTORC1 regulates translation through the direct phosphorylation of 4E-BP (translation initiation factor 4E binding protein), which binds and inhibits the initiation factor 4E and phosphorylation of S6K1 and S6K2 [5]. These kinases activate translation by phosphorylating protein S6, as well as a number of other proteins (SKAR, PDCD4, eEF-2K, eIF4B). The level of protein S6 phosphorylation using phospho-specific antibodies against Ser235/Ser236 is used to assess the kinase activity of mTORC1 [6].

The activity of mTORC1 is regulated by a number of various stimuli, such as growth factors, amino acids, glucose, and oxygen. Two key mechanisms are used in this regulation: a targeted modification of the components of this complex or regulation of Rheb GTPase, which directly interacts with mTORC1 and activates it when it is bound to GTP. The major Rheb GTPase regulator is a heterodimeric complex consisting of two tumor growth suppressor proteins: tuberin, containing conservative GAP domain, and hamartin, which stimulates the transition of Rheb GTPase from an active GTP-bound form into an inactive GDP-bound form. Inactivation of these proteins leads to the constitutive activation of Rheb GTPase, which activates mTORC1. As a result, the protein synthesizing activity is increased, and uncontrolled cell proliferation is observed [7].

It has also been demonstrated that tuberin and hamartin have an effect on the formation of primary cilia [8]. Interaction of Rab8 GTPase and guanine nucleotide exchange factor Rabin8 [9] activates Rab8 and promotes GDP release and GTP binding [10], which is involved in regulation of primary cilia formation.

Based on that, it was hypothesized that Rabin8 regulates Rheb GTPase, the major regulator of mTORC1. In the present study we showed that Rabin8 overexpression resulted in a decrease of mTORС1 activity, whereas downregulation of both Rabin8 and tuberin using siRNAs resulted in the activation of mTORC1. We also showed that Rabin8 protein could be co-immunoprecipitated with Rheb GTPase. Based on these data, we concluded that Rabin8 protein acts as a negative regulator of mTORC1 through binding to Rheb GTPase.

## EXPERIMENTAL

**Reagents and specimens**

HEK293 (human embryonic kidney) cells (АТСС, United States) were grown in DMEM with or without 10% fetal bovine serum (FBS) (Gibco, United States) added. Antibodies against Rabin8 protein (Proteintech, United States), tuberin (Abcam, United States), Myc, β-actin and mTOR, phosphospecific antibodies against Ser235/Ser236 in ribosomal protein S6 (Cell Signalling, United States), and rabbit IgG (Santa Cruz Biotechnology, United States) were used.

**Transfection**

Fugene 6 (Roche, United States) was used for the transfection of HEK293 cells with plasmid constructs; for the transfection with various siRNA, Trans-IT TKO reagent (Mirus, United States) was used according to the manufacturer’s protocol. HEK293 cells were transfected with pcDNA3.1 control vector, vector expressing Rabin8, pCMVTag3A control vector, or vector expressing Myc-Rheb fusion protein separately or together. Twenty-four hours after the transfection, the cells were washed twice and the medium, either with or without growth factors, was added. Twenty-four hours after the medium was replaced, the activity of mTORC1 was analyzed based on the phosphorylation of ribosomal protein S6 using phosphospecific antibodies against Ser235/Ser236 in protein S6. HEK293 cells were also transfected with the control, Rabin8 or tuberin siRNAs (Dharmacon, United States). Twenty-four hours after the transfection, the cells were washed twice and the medium, either with or without growth factors, was added.

**Co-immunoprecipitation**

Cells were collected in lysis buffer (Cell Signaling, United States) and cell extracts were incubated with antibodies against Rabin8 or Myc for 12 h at 4°С. The resulting complexes were precipitated by incubation with protein-A-agarose for 1 hr at 4°С, followed by centrifugation. The proteins were eluted by adding a Laemmli buffer, loaded onto denaturing gradient 4–20% polyacrylamide gel, and transferred onto polyvinyl membranes (Immobilon-P, Millipore, United States) after the electrophoresis. The membranes were blocked in a TBST buffer (137 mM NaCl, 0.1% Tween 20, 20 mM Tris, pH 7.6) (Cell Signaling, United States) containing 5% of milk for 1 h, followed by incubation with the selected primary antibodies at 4°С for a night. After the membranes were washed twice in a TBST buffer for 5 min, the corresponding secondary antibodies (Amersham, United States) were added. The membranes were washed three times in a TBST buffer for 10 min; the chemiluminescent signal was recorded by exposing with X-ray film (Kodak, United States) using a chemiluminescence kit (Perkin Elmer, United States).

## RESULTS

**Rabin8 decreases activity of mTORC1**

**Fig. 1:**
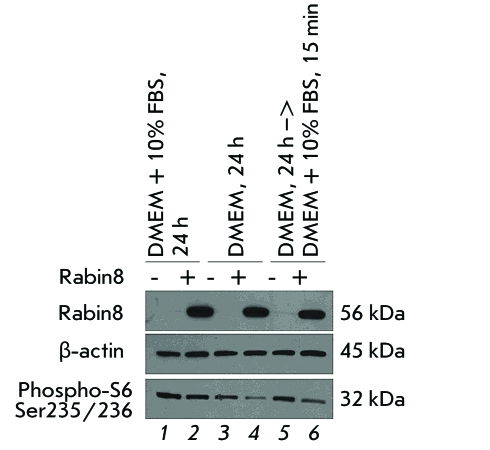
Rabin8 overexpression suppresses mTORC1 activity in HEK293 cells ( *lanes 4, 6* ). The cells were transfected with control vector pcDNA3.1 ( *lanes 1, 3, 5* ) or with Rabin8 ( *lanes 2, 4, 6* ). Activity of mTORC1 was analyzed by levels of ribosomal protein S6 phosphorylation with phospho-specific antibody against S6 (Ser235/Ser236) in the presence of growth factors ( *lanes 3,4* ), absence of growth factors ( *lanes 3, 4* ), and after 15-min stimulation of cells grown in the growth-factor-free medium with the medium containing growth factors ( *lanes 5,6* ). The levels of Rabin8 and β-аctin were measured using immunoblot analysis with specific antibodies.

The role of Rabin8 protein in the regulation of mTORC1 was studied using HEK293 cell line, the most widely used model in such experiments [11]. The cells were transfected with pcDNA3.1 control vector ( *Fig. 1, lanes 1, 3, and 5* ) or Rabin8 ( *Fig. 1, lanes 2, 4, and 6* ). In the case of the control vector, if the medium contained growth factors ( *Fig. 1, lane 1* ) mTORC1 was activated, which was measured by the phosphorylation level of ribosomal protein S6 (the bottom panel). In the absence of growth factors ( *Fig. 1, lane 3* ), mTORC1 activity was partially inhibited; the subsequent short-term stimulation of the cells after starvation with the medium containing growth factors ( *Fig. 1, lane 5* ) resulted in the complete reactivation of mTORC1. A high level of Rabin8 expression (top panel) had no effect on the activity of mTORC1 ( *Fig. 1, lane 2* ) in the medium containing growth factors; however, it resulted in a decrease of mTORC1 activity in the absence of growth factors ( *Fig. 1, lane 4* ), and after reactivation of mTORC1 with the medium containing growth factors ( *Fig. 1, lane 6* ).

**Inhibition of expression of Rabin8 or tuberin results in activation of mTORC1**

**Fig. 2:**
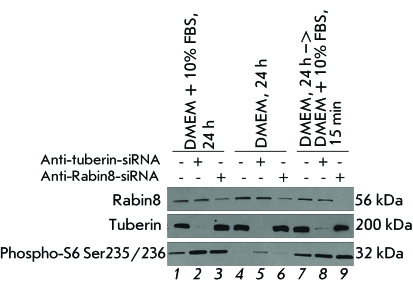
Downregulation of Rabin8 using siRNA activates mTORC1 activity in HEK293 cells ( *lanes 3,9* ), as well as downregulation of tuberin used here as a positive control ( *lanes 2,8* ). Activity of mTORC1 was analyzed in the same manner as in Fig. 1 in the presence of growth factors ( *lanes 1–3* ), in the absence of growth factors ( *lanes 4–7* ), and after 15-min stimulation of cells grown in the growth-factor-free medium with the medium containing growth factors ( *lanes 7–9* ). The levels of Rabin8 and tuberin were measured using immunoblot analysis with specific antibodies. Control siRNA – *lanes 1,4,7* .

The data obtained using over-expression of Rabin8 was confirmed using siRNAs, synthetic short RNA duplexes which initiate the targeted degradation of mRNA that is complementary to them. The following siRNAs were used as the negative and positive controls: siRNA without the complementary sequence in the human genomic mRNA (the control siRNA), and siRNA against mRNA of tuberin, the protein forming a heterodimeric complex with hamartin, as mentioned above. This complex inhibits the activity of Rheb GTPase and, therefore, the activity of mTORC1. After transfection with control siRNA ( *Fig. 2, lanes 1, 4, and 7* ), similar to the transfection with control vector (see *Fig. 1* ), the presence of growth factors in the medium ( *Fig. 2, lane 1* ) resulted in the activation of mTORC1. In the absence of growth factors ( *Fig. 2, lane 4* ), mTORC1 activity was inhibited. A short-term (15 min) stimulation of the cells grown in the medium without growth factors reactivated mTORC1 ( *Fig. 2, lane 7* ). The decrease in the expression of both tuberin and Rabin8 proteins that was observed after transfection of siRNAs against mRNA of these proteins stimulated mTORC1 activity in the presence of growth factors ( *Fig. 2, lanes 2 and 3* ). In the absence of growth factors, the activity of mTORC1 in the cells transfected with siRNA against tuberin mRNA marginally increased ( *Fig. 2, lane 5* ). The activity of mTORC1 remained unchanged after the transfection of siRNA against Rabin8 mRNA ( *Fig. 2, lane 6* ), in comparison with the transfection of the control siRNA; mTORC1 activity was higher than in the control after reactivation with the medium containing growth factors ( *Fig. 2, lanes 8 and 9* ).

**Co-immunoprecipitation of Rabin8, Rheb, and mTOR proteins**

**Fig. 3:**
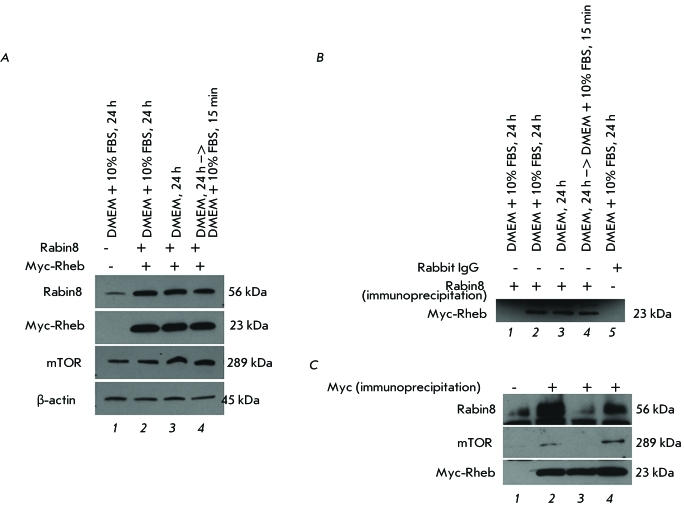
Co-immunoprecipitaion of Rabin8 and Rheb overexpressed in HEK293 cells after transfection with proper vectors. The levels of Rabin8, tuberin, mTOR and β-аctin were measured using immunoblot analysis with specific antibodies. (a) The expression levels of Rabin8, Myc-Rheb, and mTOR after co-transfection with control vectors pcDNA3.1 and pCMVTag3A ( *lane 1* ) or Rabin8 and Myc-Rheb ( *lanes 2–4* ) in the presence of growth factors ( *lanes 1,2* ), absence of growth factors ( *lane 3* ), and after 15-min stimulation of cells grown in media without growth factors with media containing growth factors ( *lane 4* ). (b) Co-immunoprecipitation using the lysates from Fig. 3a with antibodies against Rabin 8 ( *lanes 1–4* ) or control rabbit IgG ( *lane 5* ) and immunoblot analysis with antibody against Myc. The lysate from Fig. 3a ( *lane 2* ) was used for the co-immunoprecipitation with control rabbit IgG antibody. (c) Co-immunoprecipitation using the lysates from Fig. 3a with antibodies against Myc and immunoblot analysis with antibody against Rabin8, mTOR, and Myc.

As follows from the obtained results, Rabin8 is a negative regulator of mTORC1. We studied the possibility of interaction between Rabin8, Rheb, and mTOR using transfection in HEK293 by simultaneous transfection of pcDNA3.1 and pCMVTag3A control vectors ( *Fig. 3А, lane 1* ) or two vectors expressing Rabin8 and Myc-Rheb fusion protein ( *Fig. 3А, lanes 2* – *4* ) in a medium with ( *Fig. 3A, lanes 1 and 2* ) or without growth factors ( *Fig. 3A, lane 3* ), as well as after short-term stimulation of the cells grown for 24 hr without growth factors ( *Fig. 3А, lane 4* ). In order to study the interaction between Rabin8 and Rheb, the co-immunoprecipitation of cell lysates with antibodies against Rabin8 was carried out, followed by immunoblotting with antibodies against Myc epitope of the Myc-Rheb fusion protein ( *Fig. 3B* ). It was found that the interaction of Rheb with Rabin8 was independent of the presence of growth factors in the medium ( *Fig. 3B, lanes 2–4* ). Rheb was not detected after immunoprecipitation with antibodies against Rabin8 in the control lysate without expression of Myc-Rheb ( *Fig. 3B, lane 1* ) or in the lysate in which non-specific control rabbit IgG was used for co-immunoprecipitation ( *Fig. 3B, lane 5* ). In order to confirm the interaction between Rabin8 and Rheb proteins, reciprocal co-immunoprecipitation was carried out. Cell lysates were incubated with antibodies against Myc epitope of Myc-Rheb fusion protein; antibodies against mTOR and Rabin8 were used for the subsequent immunoblotting. As expected, Rheb and mTOR interacted in the presence of growth factors in the medium ( *Fig. 3B, lanes 2 and 4* ). Rheb and Rabin8 interacted in the medium containing growth factors as well ( *Fig. 3B, lanes 2 and 4* ); however, neither interaction between Rheb and Rabin8 nor between Rheb and mTOR was detected in the absence of growth factors ( *Fig. 3B, lane 3* ).

## DISCUSSION

In the present study we first demonstrated that Rabin8 regulates phosphorylation of Ser235/Ser236 in ribosomal protein S6. It has been previously shown that Ser235/Ser236 is phosphorylated by protein kinase S6K1 as a result of the activation of mTORC1, whereas the inhibitor of mTORC1, rapamycin, completely blocks phosphorylation of these residues under any conditions [2]. In accordance with this, the phosphorylation level of Ser235/Ser236 in protein S6 can be used as a convenient indicator of mTORC1 kinase activity. The obtained data suggest that Rabin8 regulates the activity of this complex. However, the role of the phosphorylation of protein S6 in the regulation of protein synthesis has not been completely determined. Transgenic mice were generated in which all amino acid residues in protein S6 were replaced with nonphosphorylatable alanine residues; however, the level of protein synthesis in different cell types in these mice remained the same as in wild-type mice [12].

We determined the phosphorylation of Ser235/Ser236 in protein S6 under various conditions, such as after cell growth in the medium containing growth factors (DMEM + 10% FBS), after growing the cells in the medium without growth factors (DMEM), and after short-term stimulation with growth factors (DMEM –> DMEM + 10% FBS) of the cells grown in the medium containing no growth factors. Since the regulation of mTORC1 is performed at several levels, the use of different growth conditions makes it easier to better understand the possible regulation mechanisms [13]. In all cases, we used DMEM medium containing amino acids, since the absence of amino acids results in the complete inhibition of mTORC1 regardless of its negative regulator tuberin [14]. The absence of growth factors activates GAP protein tuberin, which stimulates the transition of Rheb GTPase from the active GTP-bound form into inactive GDP-bound form, resulting in the inhibition of mTORC1 [15, 16]. Under these conditions, any changes inhibiting the GAP activity of tuberin or stimulating the transition of Rheb GTPase into an active form will activate mTORC1, which was indeed observed.

We also used the short-term stimulation of the cells grown in a growth-factor-free medium by growth factors to differentiate the changes in the activity of mTORC1, which occur at different rates. We found that if cells grow in a complete medium containing growth factors, the decrease in Rabin8 expression stimulated mTORC1 activity; however, Rabin8 overexpression was not sufficient to inhibit this complex. In a medium without growth factors, the decrease in Rabin8 expression was insufficient for the reactivation of the mTORC1; however, Rabin8 overexpression enhanced the inhibition of mTORC1. Rabin8 overexpression decreased the reactivation of mTORC1 resulted from the short-term stimulation by the medium with growth factors, while the decrease in Rabin8 expression stimulated reactivation. These results suggest that Rabin8 suppresses the activation of mTORC1 by growth factors.

We demonstrate for the first time that Rabin8 is bound to the major regulator of mTORC1, Rheb GTPase, which is important for the understanding of the regulation mechanism of mTORC1 by Rabin8 protein. However, it is possible that Rabin8 may interact with other components of mTORC1 as well, since we detected the catalytic subunit mTOR in complex with Rabin8 and Rheb after immunoprecipitation.There is also a possibility of mediated interaction between Rabin8 and Rheb via an unknown protein or tuberin/hamartin. However, considering that both Rab8 and Rheb belong to the family of GTPases and participate in the regulation of primary cilia formation [9], we suppose that Rabin8 has an effect on the activity of mTORC1 via Rheb GTPase. Co-immunoprecipitation experiments support this assumption, since no attenuation of the interaction between Rabin8 and Rheb proteins was detected in the growth-factor-free medium when using antibodies against Myc epitope in Myc-Rheb protein. Meanwhile, the interaction between Rheb and mTOR was abrogated, which agrees with previously published data [1]. No interaction between Rabin8 and Rheb proteins was detected in the reciprocal co-immunoprecipitation experiments for Rabin8, which can account for the different affinities of antibodies towards these proteins.

The possibility of interaction between the Rabin8 and Rheb proteins suggests a novel Rheb-dependent mechanism of regulating the primary cilia formation, which is independent of the activity of mTORC1 [8]. According to this assumption, after interaction between Rheb and Rabin8, redistribution of the Rheb function from the regulation of mTORC1 to the regulation of primary cilia formation occurs. The link between the disruption of the ciliogenesis function and various diseases isolated into a separate group of ciliopathies has recently been established. In particular, this group includes such diseases as polycystic kidney disease, the Bardet-Biedl syndrom, etc. [17]. The link between the disruption of ciliogenesis and obesity has recently been revealed [18]. In addition, primary cilia regulate the activity of the Hedgehog and Wnt signaling pathways, and disruption of their activity is involved in tumor development in various organs [19]. It should also be mentioned that activation of mTORC1 is observed in many types of tumors [20] and is required for their progression. Therefore, understanding of the regulation mechanisms and the relationship between the mTORC1 complex and the processes of primary cilia formation should result in the emergence of new approaches to the treatment of ciliopathies, obesity, and oncological diseases. 

## Abbreviations

DMEM: Dulbecco’s Modified Eagle’s Medium
FBS: fetal bovine serum
GAP protein: GTPase-activating protein
mTOR: mammalian target of Rapamycin
Rheb: Ras homologue enriched in brain
siRNA: small interfering RNA
TBST: Tris-Buffered Saline and Tween 20
